# Myopia: its historical contexts

**DOI:** 10.1136/bjophthalmol-2017-311625

**Published:** 2018-02-03

**Authors:** Paulus T V M de Jong

**Affiliations:** 1 Department of Retinal Signal Processing, Netherlands Institute for Neuroscience, Amsterdam, The Netherlands; 2 Department of Ophthalmology, Academic Medical Center, Amsterdam, The Netherlands; 3 Department of Ophthalmology, Leiden University Medical Center, Leiden, The Netherlands

**Keywords:** history of myopia, accommodation, myopia classification

## Abstract

Worldwide, and especially in Asia, myopia is a major vision-threatening disorder. From AD 1600 on, to prevent myopia, authors warned against near work without sufficient pauses. There was an abundance of theories about the causes of myopia, the most common one being the necessity of extra convergence on nearby work with thickened extraocular muscles and elevated intraocular pressure. Ocular tenotomies against myopia were in vogue for a while. Axial lengthening of the eye in myopia was mentioned around 1700, but it took 150 years to become accepted as the most prevalent sign of high myopia. In 1864, a lucid concept of myopia and other ametropias arose through a clear separation between accommodation and refraction. Posterior staphyloma was known around 1800 and its association with myopia became evident some 30 years later. There still seems to be no generally accepted classification of myopia and particularly not of degenerative or pathologic myopia. This review focuses on myopia from 350 BC until the 21st century and on the earliest writings on the histology of eyes with posterior staphyloma. A proposal for myopia classification is given.

## Introduction

The first issues of the *British Journal of Ophthalmology* contain many articles on the Great War.^1^ Thus, one can read: “Some military authorities hold that a man, unless he is a sniper, need not see what he shoots at as long as sufficient visual acuity enables him to fire in the right direction”.^2^ In the early years of this war, many men with insufficient vision were not enlisted, but in 1918 it was written: “men who require glasses have had to be enlisted, and glasses are being issued to those who need them”.^2^ These glasses were only fitted for the right eye because “Musketry instructors consider training such men (shooting from the left shoulder) more trouble than it is worth”. From a statistical point of view, few men who received such a glass would have been myopic.^3^

**Figure 1:**
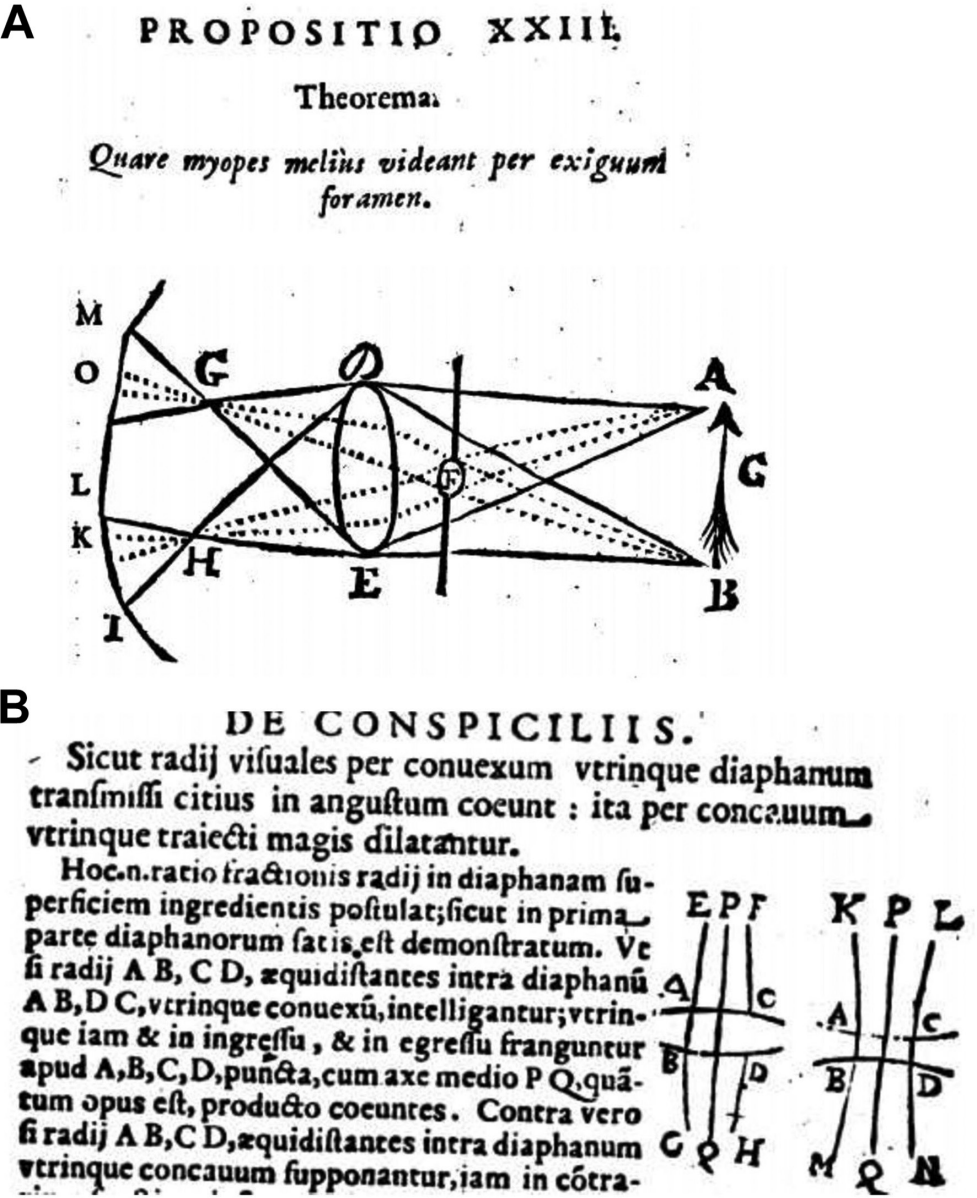
(A) Explanation of Claudius Dechales concerning how a pinhole improved far vision in his myopic eye. Without a pinhole, the image covers the area on the cornea with a diameter MI, with a pinhole KO and thus the image seems smaller with a pinhole. “A pinhole can also improve vision in elderly persons even when their rays converge behind the retina. By squeezing the eyelids, the vertical distortion is more diminished than the horizontal one. Because some lashes are placed in front of the pupil, multiple horizontal pinhole images are formed, of which often one seems the clearest”.^6^ (B) The beam path through a convex (left) and concave lens (right).^28^

The concept of myopia originated with Aristotle (350 BC), who used for the first time the word μύώψς (muoops) derivated from μύειν (muein, to close) and ωψ (oops, the eye).^4^ Aristotle made the link between bulging eyes, frequent blinking, squeezing of the eyelids, close reading and micrography.^5^ It was two millennia before it was explained why myopes see better through a pinhole and how, by squeezing the eyelids, only the vertical component of diffusion circles disappears so that horizontal lines are better seen (figure 1A).^6^ Aristotle thought that eyes deeply located in the head had better distant vision than protruding eyes. Protruding eyes could not collect well the ether movements coming from objects to the eye because they lacked the protection of the upper orbital ridge, and this could be improved by looking at distant objects through a hollow pipe.^7^ Was this a first attempt at improving visual acuity by using a pinhole? The symptoms of hyperopia and presbyopia were known shortly after Aristotle’s time, in which period vision loss was explained by defects either in the emanation theory (light emitted from the eye), the undulation theory (light from objects reaching the eye) or in the eye itself.^4^ Around 1100, this undulation theory was proven.^8 9^ The noun myopia, in Latin lusciositas, appeared as such around AD 550 in Aetius’ writings.^4 10^ Today, myopia can be defined as a refraction anomaly of the non-accommodated eye with a spherical equivalent of –0.5 dioptre (D) or more negative. Our present definition of the dioptre as a unit of refraction was, by the way, accepted only in 1872 after much lobbying during the previous World Congress of Ophthalmology.^11^ Only in 1864 was accommodation clearly separated from refraction anomalies, and a clear concept of myopia was introduced.^12^

There are several subdivisions of myopia according to the amount of refraction, age of onset and aetiology or its effects on the eye.^13–15^ Even Duke-Elder showed concerns about myopia classification at the start of his section on ‘Pathological Myopia’ where he did not know what name to choose: progressive, high, malignant or degenerative myopia.^16^ He defined degenerative myopia as “that type of myopia which is accompanied by degenerative changes occurring especially in the posterior end of the globe”. Sir Stewart wrote that myopic eyes should not be classified by their amount of objective myopic refraction (retinoscopy), and he kept the criteria for degenerative myopia vague. It is noteworthy that we write about primary versus secondary open angle glaucoma and about secondary retinal degeneration^17^ but not about primary and secondary myopia. Only Curtin named pathologic myopia, secondary myopia.^18^ The term ‘primary’ is often used to hide our ignorance about pathogenesis. When one accepts primary myopia in essence to be myopia due to elongation of the visual axis of the eye, not in conjunction with systemic syndromes involving the eye, OR due to unknown aetiology, we could call all other causes of myopia secondary myopia. ‘Primary’ myopia is commonly divided into simple and pathologic myopia (box 1) where the amount of myopia, often set at ≤−8.00 D, is a cut-off point.^16 19^ Examples of secondary myopia include myopia induced by cataract, drugs, eye drops as pilocarpine, diabetes mellitus, oxygen toxicity after diving or myopia associated with systemic syndromes. Pseudomyopia may be due to ciliary or accommodation spasm,^20^ and night myopia should be included in pseudomyopia (box 1).^21^ The highest degree of myopia determined with retinoscopy ever published is –60.0 D and the fundus of this eye showed only ‘a very small myopic crescent’.^22^ In this review, the focus will be on concepts of myopia, its causes and some proposed cures, from the beginning of our era until the 21st century. At the end, early papers on the histology of highly myopic eyes will briefly be mentioned.

Box 1 **Proposal for classification of myopia based on magnitude and cause of myopia**

- Myopia is a refraction anomaly of the non-accommodated eye with a spherical equivalent of −0.5 dioptre (D) or lower.
- Primary myopia: due to elongation of the visual axis* OR a combination of primary and secondary myopia OR unknown causes†
  - Simple myopia: myopia up to −7.75 D‡
  - High myopia: refraction ≥−8.00 D or more negative‡
- Secondary myopia: due to too strong refractive ocular media, among others
  - Corneal curvature too steep*
  - Lens swelling, higher refractive index or cataract
  - Lens dislocation
  - Medicine (eye drops) use
  - Systemic diseases including genetic syndromes involving the eye
- Pseudomyopia
  - Accommodation spasm
  - Night myopia

*More than 2 SD of the statistical average according to age, sex and ethnicity.†In daily practice, it is often not possible or practical to determine axial length. When no signs of secondary myopia are present, and essentially the cause is unknown, it is also called primary myopia.‡−8.00 is an arbitrary and often used cut-off point.

## Historical concepts of myopia until the 21st century

Only concave glasses correct myopia, and thus our focus on dating the first glasses will be on these. Emperor Nero living around AD 60 was said to be myopic because he frequently blinked when he wanted to see something^23^ and it is claimed he watched gladiator fights through a concave emerald.^24^ It is highly unlikely that concave emeralds existed at that time and Nero probably used the emerald due to the presumed soothing effect of the green reflections from it; so some authors question this emerald story.^4 5^ Green plants contain much fluid. Fluid was necessary for vision according to Aristotle and so the colour green became associated with good visual acuity.^7^ The earliest glasses, convex and concave, appeared between 1280 and 1311, and friar Bacon is credited with their introduction.^25^ In a theological tract of 1458, convex and concave beryls were mentioned^26^ and Rafael painted Pope Leo X with a concave glass in 1517^27^; thus, concave glasses were probably known from the 13th century onwards.

In 1554, Maurolycus was the first person to differentiate refraction of the eye in short vision (myopia) that could be corrected by concave lenses and long vision that needed convex lenses (figure 1B).^28^ Maurolycus considered myopia to be due to an excessively bulging lens that also should reverse an image twice so that it would appear upright on the retina.^28^ Kepler is accredited with expanding the views of Maurolycus on myopia and hyperopia.^29^ He stated, however, that eyes that see sharp both far away and close by (what we now call accommodation) have to be variable in shape and that the retina has to change its position. Kepler himself was myopic but overlooked the possibility of accommodation in myopes. He postulated that images on the retina were inverted^30^ and also wrote on the influence of normal or nearby work on the refraction of the eye. Huygens was aware, contrary to Maurolycus, that we perceive upright the inverted image on the retina but had no idea how this could work, and Scheiner soon proved this inversion.^31^ Huygens considered myopia to be due to too much convexity of the eye ball and again described how concave lenses could solve this problem.^32^ However, for centuries, concave glasses for myopia remained controversial among "ophthalmologists". Even Von Graefe warned in 1854 that they aggravate the myopic structure of the eye and obstruct the circulation.^33^

Around 1720, Boerhaave mentioned excessive length of the eye as a cause of myopia.^34^ This length could increase through infections or compression by tumours. He lectured that all male babies are myopic because their corneas are stronger curved than in female babies and that children with long heads in their youth were myopic. People who were myopic in youth see better at higher age because age makes all fibres dryer and contracts them, and the same changes occur in the cornea flattening its round shape.^34^ Was this a first attempt to explain emmetropisation? Bartisch who probably did not know of Maurolycus’ work warned in general against the use of glasses for poor vision. He did not mention refraction anomalies, let alone myopia, and advised against attentively looking at small things or reading fine script^35^ as did Saint Yves.^36^ At the beginning of the 19th century, it was pointed out that myopia was much more prevalent in higher social classes,^3^ but this was contradicted until 1883.^13^ Smoking became a risk factor for myopia, and it was considered both terrible and ridiculous to see the movements that a highly myopic (pipe) smoker makes while reading large books.^37^

The too early refraction of light rays creating myopia was considered to be due to unusual vital turgor and the special denseness of the cornea, the lens or the whole eyeball.^38^ The turgidity could be due to strong blood congestion in the head, pregnancy, experiencing a long delivery or obstipation. Axial lengthening was also attributed to the continuous contraction of both oblique eye muscles when children’s nurses hold toys very close in front of the face of small children.^38^ Beer moreover mentioned that an unusual acquired dilatation of the pupil or congenital faulty formation of the eyeball induced myopia. After lengthy considerations about the harm of concave glasses, he discussed in 1817 extraction of a clear lens in high myopia on at least one eye as a solution for myopia.^38^ His contemporary, Walther, found that myopia occurred more in children, more often in brown-haired persons than in blond ones, and that hereditary myopia was not rare. He wondered if lens fragmentation should be preferred above Beer’s lens extraction in high myopia^39^ and others opposed lens extraction altogether due to its uncertain outcome.^40^ Arnold mentioned that paralysis of the extraocular muscles causes presbyopia, but their spasm myopia. He thought that Treviranus, who said that this spasm pulls the eye back towards the bottom of the orbit, was wrong and that the straight muscles exert a slight pressure on the most curved and very thin part of the sclera.^41^ Around that same time, myopia was no longer considered a disorder but a relative state of health. ‘It relates to a normal eye like a small person to a large one’.^42^ “The most common cause of myopia is a congenital and hereditary defect. Children from myopic parents usually become myopic too. Myopia restricted to gender is not rare; for example in a family all daughters may be myopic and the sons far-sighted, and vice versa. It is of the utmost importance in raising children to switch their work far away and nearby to prevent myopia. Myopia in adults is not curable. They have to use appropriate glasses”.^42^

In 1825, Purkinje who was myopic read in an anonymous book that putting a bag containing iron filings on the eye reduced myopia due to its magnetic force. Purkinje thought that this bag worked due to its weight. At night, he put a leather pouch with 1/2 pound weight on the eye and indeed could read less but see better far away for a few hours the next morning.^43^ Ruete mentioned that myopia is often accompanied by (convergent) squint. The shortened or excessively strong inner muscles should be cut so that the straight outer muscles obtain more force. Thus, the patient can easily and voluntarily converge the visual axes in order to see far objects.^44^ He considered anisometropia either congenital or acquired by reading, drawing, embroidering or looking through a microscope or a magnifying glass. Ruete recommended Berthold’s Myopodiorthicon, which would strengthen incompletely gone adaptation facilities.^44^ This apparatus, which involves a system for progressively moving a book backwards while reading, seems to have had little effect according to others.^45^ Opaque media were found in young eyes and Arlt concluded in 1854 that these opacities led to myopia.^45^ This might be the first time that ocular media opacities inducing myopia were mentioned. Arlt remarked that myopia was more common in civilised countries and without doubt was most common in persons who from early youth on had to observe small objects while reading, writing, drawing, embroidering or sewing. Other causes of myopia were excessively high tables for children, poor lighting, pale ink, too little space between letters, too dark paper, excessively long and monotonous work without variety or pauses, and infectious diseases such as measles, smallpox, typhoid or scarlet fever. Only highly myopic persons will decide to have one or more eye muscles cut, a controversial therapy that according to Arlt had hardly any effect. He also did not expect that a bag with iron filings put on the eye of a supine patient would have any effect, given the lack of effect of this treatment in keratoconus, despite repeated paracenteses.^45^ More reports appeared on myopia due to pannous keratitis and diffuse corneal opacities but as the cause for this myopia, again, holding objects close to the eye due to the opacities was mentioned.^33^ Von Graefe wrote that myopia was based on elongation of the eye axis and not, as he had formerly thought, on a change in refraction of the vitreous fluid.^46^ He wrote this over 100 years after Boerhaave’s and 50 years after Beer’s writings, and at a time when this was well known in the UK^47^ where Von Graefe had good connections. So for over 300 years from Bartisch’s time onwards, authors disagreed about whether myopia was due to near work and about whether it was restricted to the lower social classes. Part of this disagreement was resolved by comparing 10 000 army recruits of whom 12 were rejected due to myopia with 127 Oxford students of whom 25% used glasses.^3^ Similarly, over 7500 conscript soldiers from Copenhagen or the countryside, who had detailed job descriptions, were classified as performing near work or no near work. Myopia was diagnosed as ≥−2.00 D and 18% of the near workers were myopic versus 4% in the other group.^13^

**Figure 2:**
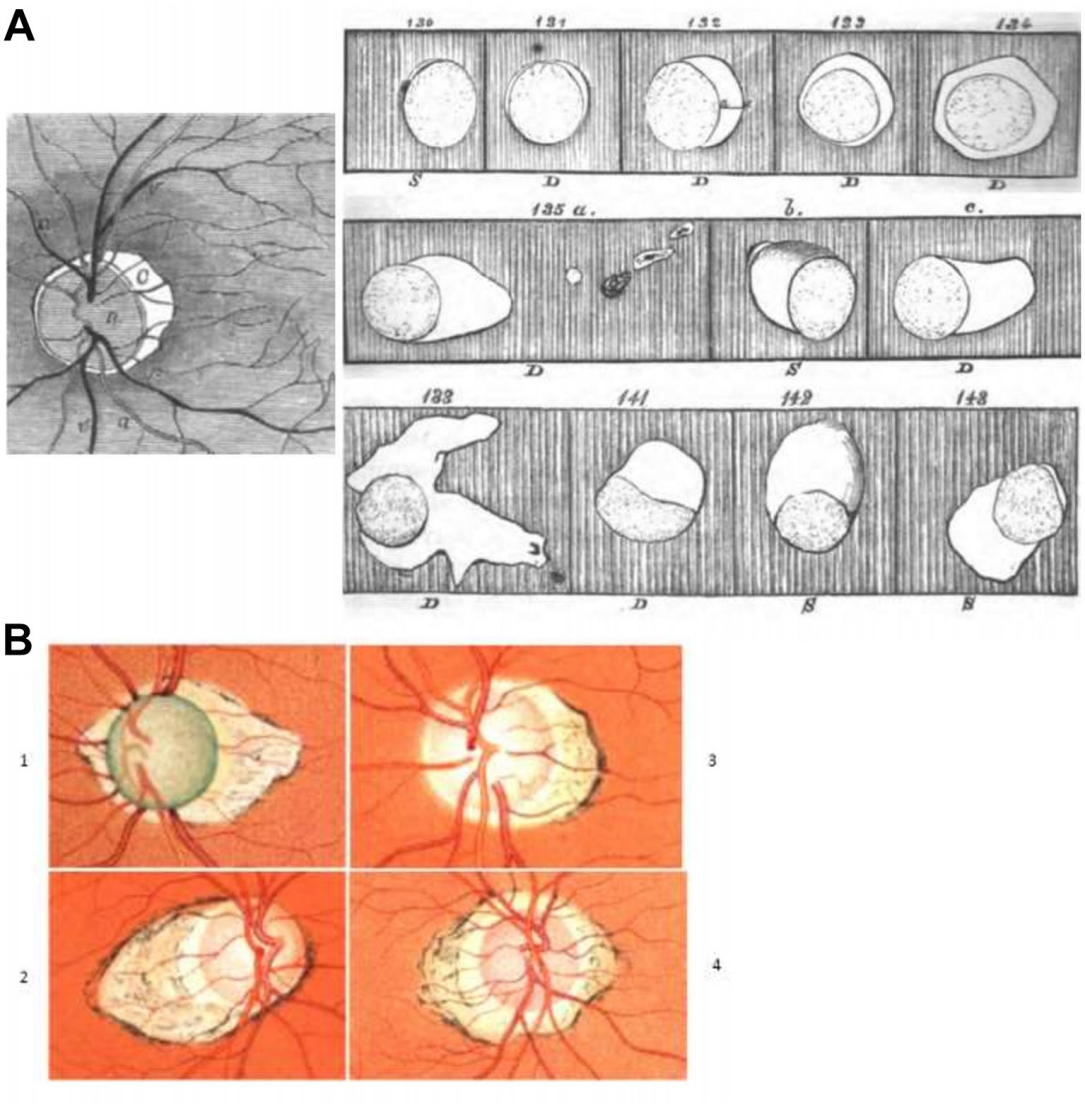
(A) Crescentic, strongly reflecting surface C, immediately distinguishing myopia on ophthalmoscopy.^48^ On the right, a selection of sketches by Donders of the various types of cones he saw in 1500 myopic patients, published 13 years after the invention of the ophthalmoscope.^49^ (B) 1. Glaucomatous optic nerve excavation and posterior staphyloma. 2. Medium-sized ‘conus’ in an eye that was myopic due to posterior staphyloma. 3. Very large conus in an eye that was myopic due to posterior staphyloma. 4. Double conus in an eye that was myopic due to posterior staphyloma. 53

Donders introduced the concepts of emmetropia, hyperopia, presbyopia and ametropia after clearly distinguishing accommodation from refraction.^12 48^ He considered himself primarily to be a physiologist, and he showed that the corneal curvature did not essentially differ between ametropic and emmetropic eyes. In extreme myopia, it became flatter. ‘Intra-individual variation in corneal curvature is small and women have a shorter radius than men’. Although this had been hinted at earlier,^25^ Donders clearly stated for the first time after examining over 2500 patients that also a myopic eye can become presbyopic. ‘Myopia is almost always somewhat progressive, and this is the rule between the ages of 15 and 25 years’. As causes of axial elongation, he mentioned pressure of the eye muscles on convergence of the eyes, elevated intraocular pressure by blood congestion in the stooping position, congestive processes in the ocular fundus or a combination of these.^48^ Elongation occurred especially in the posterior pole due to lack of supporting muscles. ‘Watchmakers have no myopia because they look with only one eye and thus have little convergence and no stooping position’. He found no cases with extraordinary convexity of the crystalline lens. Donders stated that a myopic eye is not a healthy eye and that cases of myopia were much more common in his private practice than in his hospital for needy eye patients. Nine years after the invention of the ophthalmoscope,^49^ after repeatedly examining 1500 myopic eyes and drawing 700 in detail, he wrote that myopia progression was faster the higher the amount of myopia in the eye. In high myopia, atrophy of the membranes develops, leading to posterior chorioscleritis as identified by Von Graefe.^12^ Peripapillary atrophy was quite common in myopia (figure 2A). Donders wrote “It is not rare that in 60–70 year old persons, if not much earlier, vision is irretrievably lost by either separation of the retina and choroid, by haemorrhage, or atrophy and degeneration of the macula lutea”.^12^ In a later publication, he added: “I have seen in several myopic macula’s irregular grainy pigment distribution with a bluish and elevated spot, sometimes the size of the disc, possibly connected to the haemorrhage that I have seen several times restricted to a part of the fovea”.^9^ Förster attributed the black spot in the macula of myopic eyes to shrinking of the retina towards the macula. He mentioned exudation but never haemorrhages.^50^ Pictures of retinal haemorrhages in high myopia appeared 25 years later.^51^ So if we still stick to eponyms, should Förster’s or Fuchs’s spot^52^ not be called Donder’s spot?^9^

Thus, in around 1850, peripapillary atrophy, which is common in myopia, was named posterior ‘Sclerotico choroideitis’ by Von Graefe and was found in 9 out of 10 highly myopic persons. Doubts about this name were voiced because no signs of inflammation were found.^33^ Later, Von Graefe wrote that the posterior chorioscleritis was nothing other than the posterior staphyloma of Scarpa. With his first ophthalmoscopic observation, Donders recognised myopia with high certainty, by a semilunar stark reflecting peripapillary area (figure 2A). He described in detail peripapillary atrophy in myopia at various ages and confirmed an earlier observation^20^ that its largest diameter was perpendicular to its axis. Donders mentioned that in rare cases the atrophic area was markedly excavated, as seen by a certain inclination of the retinal vessels. This was probably one of the earliest descriptions of scleral ectasia by ophthalmoscopy, and shortly afterwards a colour image of scleral ectasia appeared (figure 2B).^53^ Peripapillary atrophy in posterior sclerectasia was sometimes surrounded by an area in which the choroidal tissue was strongly pulled apart and partly deprived of its stromal pigment.^20^ Was this an early description of what we now call zone α? The peripapillary atrophy was later subdivided in zone α, farthest away from the disc and containing Bruch’s membrane (BrM) plus an irregular retinal pigment epithelium (RPE). An intermediate zone β contained BrM without RPE, and zone γ was closest to the disc having no BrM. Zone β was associated both with increasing glaucomatous nerve fibre loss and myopia, but not with axial elongation. Axial elongation was associated with zone γ. Recently, a zone δ was added, the inner part of the γ zone in the region of the elongated and thinned peripapillary scleral flange, that covers the orbital cerebrospinal fluid space.^54^ This addition was, among others, based on several studies using optical coherent tomography. There seems to be no histology of myopic eyes in which during life, detailed tomography had been performed of these zones.

## Histology of myopic posterior staphyloma

**Figure 3:**
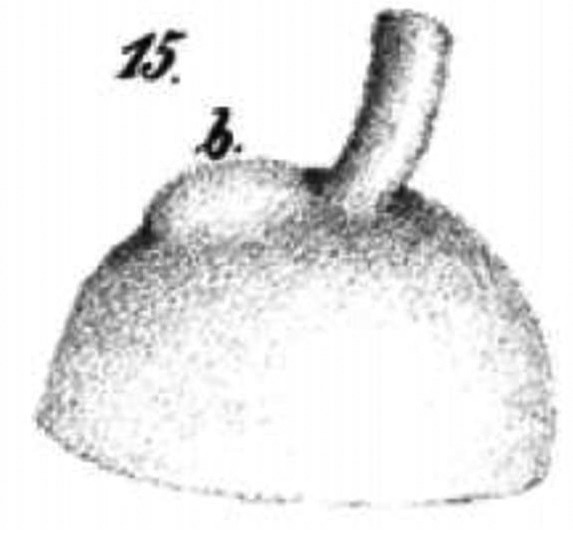
Scleral protuberance (b) in a sheep fetus^58^ which would be the weak spot leading to posterior staphyloma.^56^

In 1801, Scarpa was the first to describe the gross anatomy of unilateral posterior staphyloma in a 35-year-old and a 40-year-old woman.^55^ Lateral to the optic nerve, a tumour-like swelling, the size of a hazelnut, was present. The vitreous was disorganised and contained clear water. ‘In the excavation of the staphyloma, the white nerve smear of the retina was lacking’. The thin choroid contained no colour and the sclera was paper-thin. Three more cases with posterior staphyloma, named posterior hydrophthalmus by the author, appeared.^56^ The eyes were up to 2 inches long, had very thin posterior sclerae with bluish discolouration and sometimes lacked retinal and choroidal tissues. The posterior staphyloma had nothing to do with choroidal varices and the staphyloma started near the scleral protuberance, visible at a fetal age of 4 months (figure 3).^57 58^ No myopia was mentioned by Scarpa nor by Von Ammon, and the first to do so in both staphylomatous eyes of a printer was Ritterich.^59^ Three years later, both eyes of a 76-year-old blind woman were described with a bilateral blue scleral staphyloma.^60^ The refraction was unknown and the thinned sclera and choroid had partly grown together. The ciliary body was partly gone, as were the ciliary nerves in the sclera and the choroid. Arlt mentioned that a staphyloma formerly was wrongly considered to be a varix of the ciliary body and that the sclera became staphylomatous due to diverging scleral fibres and enhanced intraocular pressure. Next, he described the ocular histology of four myopic persons whom he initially classified as having the posterior staphyloma of Scarpa but that he later associated with myopia, after hearing Ritterich’ s presentation. The internal rectus and inferior oblique muscles were remarkably thick. In the most myopic eye, he saw marked thinning of the choroid and spotty brown pigment with the largest spot exactly in the macula.^45^ Around that time, Von Graefe described autopsy findings in two eyes with posterior sclerotic choroiditis that he also could examine with an ophthalmoscope. The visual axis of the eyes was 29 and 30.5 mm long. On histology, the retina was intact over a thinned or absent choroid with sparse pigment. The ciliary vessels were completely obliterated.^33^ One more publication on a very much enlarged eye of a 69-year-old woman with a large ‘Staphyloma (verum Scarpae)’ was published.^61^ Its axial length was 32 mm. Cross-sectioning of the staphyloma revealed marked thinning of the outer eye coverings. There was normal retinal layering but choroidal atrophy, especially of the capillary layer. The retinal thickness in the staphyloma was 0.096 mm, the choroidal thickness 0.044 mm and the scleral 0.170 mm. The intervaginal space of the optic nerve formed a triangle filled with broad fibrous bands.^61^ Later on, several manuscripts on the histology of high myopia generally confirmed these results.^62 63^

Some aspects of myopia have not been addressed in this review. Other causes of myopia were incidentally mentioned (table 1) and there was no space for discussing, for example, mechanisms of emmetropisation, treatments such as scleral reinforcements or the elusive genetics of myopia. These genetics refer to population or family level studies and also to upregulation or downregulation of genes during myopia progression. Nevertheless, it is hoped that this overview will stimulate myopia researchers to select the most interesting research topics for myopia in the future.

**Table 1:** Hypotheses about aetiology of myopia

Year AD	Aetiology	First author*	References
400 BC 160	Not enough ‘pneuma’ in the eye or ‘pneuma’ coming from the eye not strong enough to penetrate the air	Plato, Galen	7
350 BC	Water content of eye too high	Aristotle	4
1583	Near work or fine script reading, insufficient lighting	Bartisch	12 29 30 35 36 38 42 45
1611	Excessive bulging lens	Maurolycus	28
1703	Excessive convexity of eye ball	Huygens	32
1720	Axial elongation, for example, due to infections or tumour compression	Boerhaave	34 45
1720	Excessive corneal curvature	Boerhaave	34
1801	Smoking	Himly	37
1813	Use of concave glasses	Ware	3 43
1813	Higher social class/education	Ware	3
1813	Loss of orbital fat leading to oval eye	Ware	3
1817	Unusual vital eye turgor (congestion of the head, in pregnancy, during delivery or due to obstipation)	Beer	12 38
1817	High density of ocular media	Beer	38
1817	Oblique muscle over action	Beer	38
1817	Unusual acquired pupillary dilatation	Beer	38
1817	Congenital faulty eyeball formation	Beer	38
1826	Monocular concave glass creates myopia in second eye	Weller	40
1830	Brown hair	Walther	39
1830	Heredity	Walther	39 42 45
1832	Spasm of extraocular muscles	Arnold	41
1845	Convergent squint due to overactive internal rectus muscle	Ruete	44
1848	External eye muscle neurosis	Szokalski	64
1850	Myopia in distans. Involuntary accommodation	Fronmüller	65
1854	Posterior choroidoscleritis; chronic inflammation	Von Graefe	33
1855	Change in vitreous refractive power	Von Graefe	46
1856	Vitreous liquefaction	Arlt	45
1856	Accommodation error	Arlt	45
1856	Opacities in ocular media	Arlt	33 45
1860	Elevated intraocular pressure due to pressure of extraocular muscles on convergence	Donders	12
1883	>−9.00D in low class women without near work	Tscherning	13
1905	Congenital defective development of scleral elastic fibres	Lange	66

*Only the author who seems to have mentioned a theory for the first time has been named. Under references, later authors with similar views are added.
